# Doppler-Guided Hemorrhoid Artery Ligation with Recto-Anal-Repair Modification: Functional Evaluation and Safety Assessment of a New Minimally Invasive Method of Treatment of Advanced Hemorrhoidal Disease

**DOI:** 10.1100/2012/324040

**Published:** 2012-04-01

**Authors:** Piotr Walega, Michal Romaniszyn, Jakub Kenig, Roman Herman, Wojciech Nowak

**Affiliations:** ^1^3rd Department of General Surgery, Jagiellonian University School of Medicine, Pradnicka Street 35-37, 31202 Krakow, Poland; ^2^Department of Experimental and Clinical Surgery, Institute of Physiotherapy, Jagiellonian University School of Medicine, Michalowskiego Street 12, 31126 Krakow, Poland

## Abstract

*Purpose*: We present 12-month followup results of functional evaluation and safety assessment of a modification of hemorrhoidal artery ligation (DGHAL) called Recto-Anal-Repair (RAR) in treatment of advanced hemorrhoidal disease (HD). *Methods*: Patients with grade III and IV HD underwent the RAR procedure (DGHAL combined with restoration of prolapsed hemorrhoids to their anatomical position with longitudinal sutures). Each patient had rectal examination, anorectal manometry, and QoL questionnaire performed before 3 months, and 12 months after RAR procedure. *Results*: 20 patients completed 12-month followup. There were no major complications. 3 months after RAR, 5 cases of residual mucosal prolapse were detected (25%), while only 3 patients (15%) reported persistence of symptoms. 12 months after RAR, another 3 HD recurrences were detected, to a total of 8 patients (40%) with HD recurrence. Anal pressures after RAR were significantly lower than before (*P* < 0.05), and the effect was persistent 12 months after RAR. One patient (5%) reported occasional soiling 3 months after RAR. *Conclusions*: RAR seems to be a safe method of treatment of advanced HD with no major complications. The procedure has a significant influence on anal pressures, with no evidence of risk of fecal incontinence after the operation.

## 1. Introduction

Hemorrhoids are normal part of human anorectum and consist of arterioles, venules, and arteriolar-venular communications supported by fibromuscular tissue [1, 2]. Surgical methods of treatment of advanced hemorrhoidal disease include classical hemorrhoidectomies (Milligan-Morgan, Fergusson), Longo-stapled hemorroidectomy, and others. Each method has its advantages and disadvantages, and method-specific complications, including anal canal strictures, sensation impairment, and sphincter damage, resulting in fecal incontinence [3–5].

In 1995, Morinaga et al. described a new method of treatment of hemorrhoidal disease, based on hemorrhoidal artery ligation, guided by a Doppler flowmeter. The aim of the new approach was to preserve hemorrhoidal plexuses and overlaying mucosa [6]. It is now a very popular method of treatment of grade II and III hemorrhoidal disease in some countries (Austria, Italy), recommended by some colorectal societies as an optimal method of treatment of these stages of HD, for its simplicity and low risk of complications [7]. The recurrence rate for grade IV hemorrhoidal disease is significantly higher in patients treated with DGHAL than with hemorrhodectomy [8], also standard DGHAL does not address the issue of prolapsed mucosa. First of mentioned problems rarely becomes an issue because of very low risk of the procedure, ability to re-apply the same method in case of lower effectiveness and a very good tolerance of this method by patients. This is why modification of DGHAL addressing the issue of mucosal prolaps is a very attractive option. This could lead to more wide use of Doppler-guided ligation also for higher grade heamorrhoids [7].

**Figure 1:**
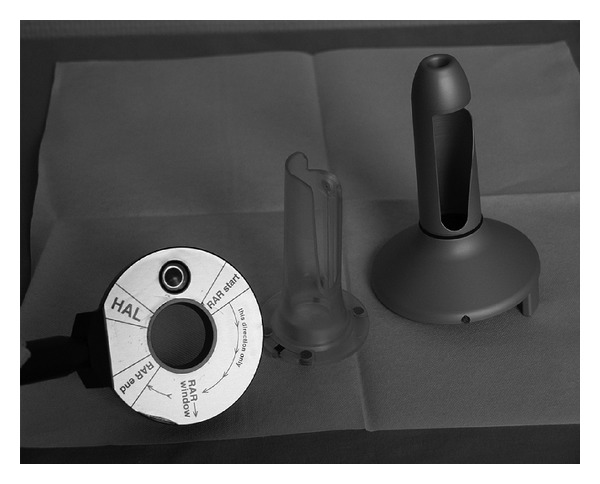
A.M.I. DGHAL-RAR Proctoscope.

The most recent modification of selective hemorrhoidal artery ligation method, the rectoanal repair, combines selective Doppler-guided hemorrhoidal arteries ligation with plication of the prolapsed rectal mucosa, using the specially designed proctoscope (A.M.I., Austria)—Figure 1. Instead of excision of the hemorrhoids, the aim of this procedure is to reduce enlarged hemorrhoids by ligation of hemorrhoidal arteries and to restore anatomical position of the prolapsed mucosa. In the 3rd Department of General Surgery, Jagiellonian University, this method is being used since 2006, as a part of a multicenter clinical study (Austria, Poland, India, Italy). 

The aim of this paper is to present this new technique and preliminary results of functional evaluation and safety assessment of RAR procedure in the treatment of IIIrd and IVth grade HD, conducted at the 3rd Department of Genetral Surgery, Jagiellonian University.

## 2. Methods

The study had been positively approved by the Bioethics Committee. 40 patients, 27 male, 13 female, of average age of 53 years (29–74 years), with symptomatic IIIrd and IVth grade HD were qualified for the study. Each patient had standardized diagnostic procedures performed: rectal examination, endoscopic and endorectal ultrasound examination, anorectal manometry, and Quality of Life questionnaires (GIQL, FIQL).

**Qualification criteria:**

- symptomatic hemorrhoidal disease with prolapsing hemorrhoids which either had to be reducible manually (IIIrd grade) or could not be manually reduced (IVth grade),
- no history of fecal incontinence prior to enrolment (based on fecal incontinence score: Fecal Incontinence Severity Index),
- no signs of sphincter damage in endorectal ultrasound examination,
- no pathological findings in a diagnostic endoscopic examination of the colon,
- patient's written consent to participate in the study.

Apart from prolapsing or permanently prolapsed hemorrhoids (100%), chief complaints on admission were bleeding (92.5%), itching (72.5%), and painful defecation (70.00%). There were no significant pathological findings in patients' functional anorectal assessments before the procedure, none of the patients reported fecal incontinence. 

**Figure 2:**
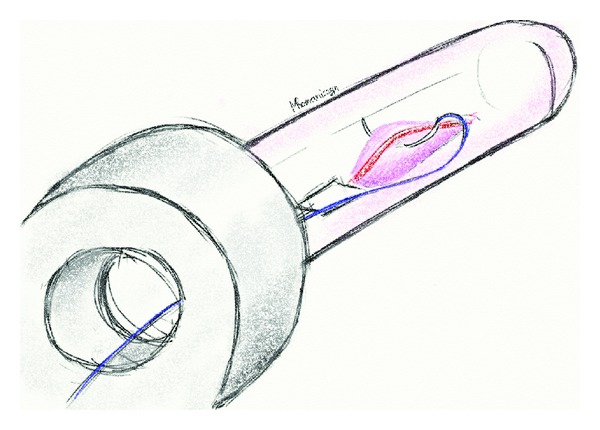
A stitch ligating a haemorrhoidal artery.

Two patients were disqualified from the procedure shortly before admission because of contraindications to anesthesia. All other 38 patients were admitted to the 3rd Department of General Surgery, Jagiellonian University for preoperative evaluation and underwent RAR procedure the next day. All patients were operated in Lloyd-Davis' position. Using a specially designed longitudinal opening in the proctoscope, continuous absorbable sutures are laid longitudinally along the anal canal to lift prolapsed hemorrhoids back to their anatomical position.The first stage of the operation consisted of standard Doppler-guided hemorrhoidal artery ligation (DGHAL) using an A.M.I. DGHAL-RAR proctoscope. The proctoscope consisted of a modified DGHAL probe and a specially designed proctoscope tube, with a 5 cm longitudinal opening. The hemorrhoidal arteries were detected with a Doppler flowmeter device built into the proctoscope and ligated with 2/0 absorbable suture (polyglycan), on 5/8 needle, using double stitch (figure-of-eight) method, as described by Scheyer et al. [8] (Figure 2). 

**Figure 3:**
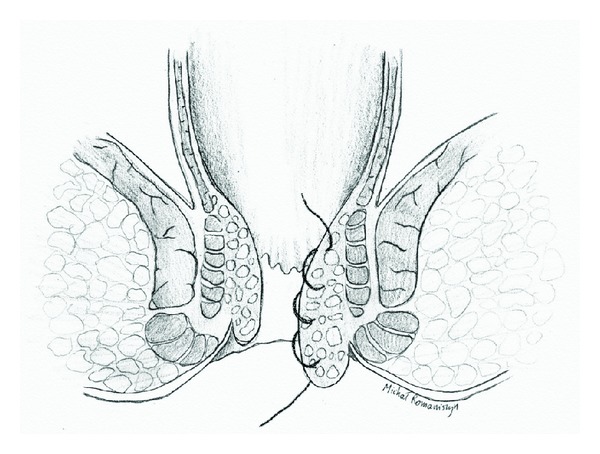
Longitudinal suture, covering the whole mass of a prolapsed haemorrhoid.

**Figure 4:**
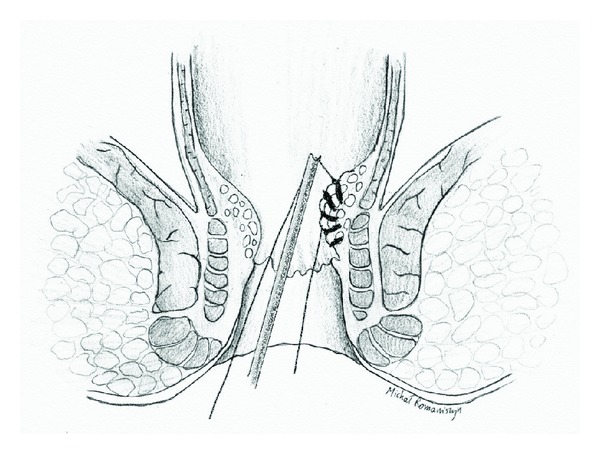
Haemorrhoid lifted back into its anatomical position by tying the suture.

After all detectable arteries were ligated (no more arterial Doppler signals could be detected), surgeon proceeded with second part of the procedure. Prolapsed hemorrhoids were identified in anoscopic examination. The proctoscope was placed in the anal canal with the longitudinal opening in “closed” position, with probe window placed over selected prolapsed hemorrhoid. By turning the probe inside the proctoscope tube, the longitudinal opening of the proctoscope was gradually opened, from proximal (cranial) to distal (caudal) part. This allowed placing a continuous suture along the prolapsed mucosa (Figure 3). The suture, covering the whole mass of a prolapsed hemorrhoid was tied, lifting the hemorrhoid back into its anatomical position (Figure 4). This procedure was repeated for each prolapsed hemorrhoid until no more prolapsed mucosa was visible outside the anal canal. 

After the operation, patients were kept in the ward for 24 hours of observation for detailed assessment of postoperative course and then discharged home. We would like to underline that prolonged hospital stay was due to our aim of careful evaluation of postoperative course during first 24 hours. For postoperative pain control, NSAIDs (ketoprofen) were administered on demand for the cumulative dose upto 300 mg daily. 

Three months and 12 months after surgery each patient had rectal examination, anorectal manometry and Quality of Life questionnaires performed again. All data, including photographical documentation was collected on standardized data forms and analyzed.

Acquired anorectal pressure data was checked for normality (Shapiro-Wilk's test) and analyzed toward significant differences using nonparametric Wilcoxon matched pairs test, as distribution of samples was not normal. The manometric data was also checked with multiple nonparametric Kruskal-Wallis ANOVA tests for any influence of disturbing factors (age, gender, etc.).

## 3. Results

Of the initial 38 patients, 18 were lost to full 12-month follow-up (mostly due to refusal of further participation in the study or incomplete manometric data) and were excluded from the analysis. Collected data of the remaining 20 patients were analyzed (Table 1). In manometric data acquired prior to operation, there was no significant correlation of either basal anal pressure (BAP) or squeeze anal pressure (SAP) levels with age or gender in study group. There was no correlation between HD grade (III versus IV) and manometric findings (*P* > 0.05). 

The RAR procedure itself took about 35 minutes on average (25–75) from beginning of anesthetical procedures to transportation of the patient back to bed from the operating table. Mean number of arteries ligated during the procedure was 5.65 (4–8), most frequently found on 1 and 11 o'clock (in Loyd-Davis' position, corresponding to 5 and 7 o'clock in supine position). There were on average 2.5 (1–4) longitudinal sutures used to pull prolapsed mucosa up into the anal canal. There were three cases (15%) of intraoperative bleeding requiring additional haemostatic sutures. Early postoperative bleeding (approximately 60 mL of blood) was reported in one patient (5%), on the first day after surgery. The bleeding was successfully managed with sterile anal tamponade (Lockhart-Mummary type). There were no other complications in perioperative period which would require surgical intervention. Postoperative pain was easily managed with NSAIDs administered i.v. or p.o. The hospital stay was standardized and lasted for a total of 48 hours, due to our aim of careful evaluation of postoperative course.

In the followup examination 3 months after RAR procedure, there were 5 cases (25%) of minor residual hemorrhoidal prolapse among the 20 patients included in the final analysis, while only 3 of them (15%) reported residual symptoms (painful defecation and itching). These 3 patients were all among the first 10, who underwent rectoanal repair in our department. There were no cases of persistent bleeding within the first 3 months after RAR procedure. 

In anorectal manometry assessment, anal pressure levels recorded 3 months after RAR were significantly lower than before the procedure (Table 2). On average BAP dropped 11.53%, SAP 12.2% in women, BAP dropped 5.82% and SAP 6.03% in men (*P* < 0.05). These results were not correlated in any way with age nor gender of the patient nor grade of hemorrhoidal disease (*P* > 0.05).

Based on the Quality of Life questionnaires (GIQL, FIQL), most of the patients were satisfied with the results of the treatment, reporting better overall wellness and self-confidence, despite of noncomplete reduction of mucosal prolapse in a few cases. Mean GIQLI preoperatively was 110 patients, while postoperatively mean GIQL index reached 135 patients, 19 patients (95%) had significant GIQLI improvement. However, one of the 20 patients (5%), 73-year-old male, reported occasional continence problems at the followup examination 3 months after the procedure (incontinence of gases, occasional soiling), with onset about one month after surgery. Manometric findings were also normal in this patient. Change of diet and simethicone administration three times a day was sufficient to relieve the patient's symptoms.

In the followup examination 12 months after RAR procedure, there were 3 new cases of hemorrhoidal prolapse recurrence, giving a total of 8 known patients with hemorrhoidal prolapse 12 months after the procedure. There were no cases of persistent bleeding, and the satisfaction level measured with Quality of Life questionnaires was still high in most of the patients (95%).

In anorectal manometry assessment, pressure levels recorded 12 months after RAR were similar to values recorded 3 months after the procedure in the same patients (*P* > 0.05). There were no new cases of functional disorders recorded, the patient who reported incontinence after the procedure reported at the 12-month followup slight improvement, with persistence of gases incontinence.

## 4. Discussion

The researchers are in a constant search of new methods of treating hemorrhoidal disease that would offer not only high effectiveness and low morbidity, but also short recovery and good postoperative comfort. Rubber band ligation used in stage II and III hemorrhoids can be complicated by postprocedure bleeding in up to 5% of cases [9]. The efficacy of this method is 76% in stage II, 66% in stage III, and less then 20% in IV degree hemorrhoids. Rubber band ligatures are placed under limited visual control, near the dentate line; heamorrhoidal arteries are left open, which results in a high probability of recurrence [10]. Baron's method also requires several applications of rubber bands since most of proctologists refrain from ligation of all hemorrhoidal piles during one procedure. Moderately invasive methods such as Longo's operation are burdened with a relatively high risk of complications, including severe complications such as perforation, occlusion of rectum, retroperitoneal hematoma, and Furnier's gangrene [11–16]. During the DGHAL-RAR procedure, all sutures are placed under direct visual control, so risk of misaligned sutures is greatly reduced. Additionally, during stapler-based procedures, a continuous ring of mucosa is being cut out, while in DGHAL-RAR, longitudinal stripes of untouched mucosa between the RAR sutures reduce the risk of impairment of anorectal function and sensation. Conventional surgical hemorrhoidectomy according to Milligan Morgan, Ferguson, and their modifications represent the most effective treatment method of HD that is currently available. However, the effectiveness of these methods is limited by various complications such as sphincter dysfunction (in up to 25% of patients), rectoanal coordination impairment due to partial resection of anal mucosa (another 10% of patients), postoperative bleeding, or infection up to 5–15% of patients [15]. Also postoperative recovery usually lasts from a few days to 2 weeks. Therefore, taking all these facts into consideration, new methods of treatment like DGHAL and RAR, aside from enhancing effectiveness, concentrate on preserving natural anatomical and histological structure of anorectal region as much as on the possibility to prevent anorectal function impairment. They are also aimed at shortening the postprocedure recovery [17, 18]. 

It is said that hemorrhoidal plexus is responsible for 15–20% of resting anal pressure. According to some studies [19–21], these pressures are significantly higher in patients with HD compared to healthy individuals, and drop after surgical management of HD regardless of chosen method (Barron, Milligan-Morgan, Longo) was also reported [19, 20, 22]. Some authors even raise a question if elevated resting anal pressure is secondary to swelling of hemorrhoidal cusions, or if it is an etiological factor of hemorrhoidal disease [19]. On the other hand, many other authors state that there was no significant change in manometric findings in patients treated for hemorrhoidal disease [23–25] leaving the question of initially raised pressure unanswered. Papers concerning the DGHAL method also report no significant changes in basal and squeeze pressures [25]. Our study shows a significant drop in resting anal pressure after RAR, with coexistent minor influence of the RAR procedure on squeeze anal pressure, although we have no proof if the pressures were elevated initially as compared to healthy individuals. A certain drawback of our study was the relatively small group of patients—this was due to strict qualification—patients operated with this method who had not met the qualification criteria were not included in the study, to keep the study group uniform concerning initial functional conditions. The other factor limiting the quantity of patients in the analyzed group was the fact that some patients refused to further participate in the study, or their manometric followup was not complete due to omitted followup visits. Taking this into consideration, there is still a need for a complex study involving an age- and gender-matched control group of healthy individuals for comparison or perhaps a large-scale prospective trial, to answer this question. 

The clinical results of the rectoanal repair procedure are very promising, as most of the patients were satisfied with the outcome, although the observation period of 12 months is too short to conclude the long-term effectiveness of the procedure. Moreover, many of the patients treated with this method in our department did not complete full followup visits plan, so clinical results in those patients are unknown. It can be only guessed that most of them did not have any symptoms after the treatment, and so did not find visiting a doctor necessary. In short-term studies concerning stapler-based techniques, the recurrence rates were lower than in our analyzed group of patients [26–29]. DGHAL/RAR however may offer better safety profile and lower risk of anorectal disturbances. Additionally, recovery after RAR procedure was much quicker compared to classical Milligan-Morgan or Ferguson hemorrhoidectomy [4].

## 5. Conclusion

Rectoanal repair seems to be a safe method of treatment of IIIrd and IVth grade hemorrhoidal disease with no major complications and a high rate of good short-term results. The procedure has a significant influence on resting and squeeze anal pressure, with no evidence of risk of fecal incontinence after the operation. It remains to be answered if that is a result of return to normal anal tone or should be considered as an adverse effect. However, this is a preliminary study with small series of patients and short followup time, so it is difficult to assess long-term efficacy, recurrence rates, and long-term influence on anorectal function, which still need to be assessed in larger studies with longer followup period and bigger groups of patients.

## Figures and Tables

**Table 1:** 

			Grade (Parks)		
	*n*	Mean age	III	III/IV	IV
Male	12	54 (29–70)	3	6	3
Female	8	56 (40–68)	6	1	1

**Table 2:** Manometric findings upon qualification and 3 months after DGHAL-RAR procedure.

	Mean BAP			Mean SAP			Physiological RAIR (before/after)	RSCC present (before/after)
	Upon qualification	3 months after RAR	12 months after RAR	Upon qualification	3 months after RAR	12 months after RAR		
Male	78.83	72.17	67.92	214.50	199.75	198.75	12/12	12/12
Female	64.50	56.50	58.75	129.38	111.25	119.38	8/8	8/8
